# Social inequalities and COVID-19 mortality between neighborhoods of Bariloche city, Argentina

**DOI:** 10.1186/s12939-023-02019-w

**Published:** 2023-09-28

**Authors:** Mónica Serena Perner, Andrés Trotta, Usama Bilal, Binod Acharya, Harrison Quick, Natalia Pacífico, Romina Berazategui, Marcio Alazraqui, Ana V. Diez Roux

**Affiliations:** 1 Institute of Collective Health, National University of Lanus, Buenos Aires, Argentina; 2 CONICET (National Scientific and Technical Research Council), Buenos Aires, Argentina; 3 Urban Health Collaborative, Dornsife School of Public Health, Drexel University, Philadelphia, USA; 4 Department of Epidemiology and Biostatistics, Dornsife School of Public Health, Drexel University, Philadelphia, USA; 5 Dr. Ramón Carrillo Hospital, Bariloche, Argentina

**Keywords:** COVID-19, Spatial analysis, Urban health, Social inequalities, Small areas

## Abstract

**Background:**

The COVID-19 pandemic has shown how intraurban inequalities are likely to reinforce health and social inequalities. Studies at small area level help to visualize social inequialities hidden in large areas as cities or regions.

**Aim:**

To describe the spatial patterning of COVID-19 death rates in neighborhoods of the medium-sized city of Bariloche, Argentina, and to explore its relationship with the socioeconomic characteristics of neighborhoods.

**Methods:**

We conducted an ecological study in Bariloche, Argentina. The outcome was counts of COVID-19 deaths between June 2020 and May 2022 obtained from the surveillance system and georeferenced to neighborhoods. We estimated crude- and age-adjusted death rates by neighborhood using a Bayesian approach through a Poisson regression that accounts for spatial-autocorrelation via Conditional Autoregressive (CAR) structure. We also analyzed associations of age-adjusted death rates with area-level socioeconomic indicators.

**Results:**

Median COVID-19 death rate across neighborhoods was 17.9 (10th/90th percentile of 6.3/35.2) per 10,000 inhabitants. We found lower age-adjusted rates in the city core and western part of the city. The age-adjusted death rate in the most deprived areas was almost double than in the least deprived areas, with an education-related relative index of inequality (RII) of 2.14 (95% CI 1.55 to 2.96).

**Conclusion:**

We found spatial heterogeneity and intraurban variability in age-adjusted COVID-19 death rates, with a clear social gradient, and a higher burden in already deprived areas. This highlights the importance of studying inequalities in health outcomes across small areas to inform placed-based interventions.

**Supplementary Information:**

The online version contains supplementary material available at 10.1186/s12939-023-02019-w.

## Introduction

By mid-2022 the Latin American region, which houses only 8.4% of the world population [1], had experienced 26.6% of the global COVID-19 deaths [2], making it one of the most affected regions by the pandemic [3]. Cities were especially vulnerable to viral spread, especially among residents living in segregated neighborhoods [1, 4, 5] due in part to working conditions, overcrowding, and poor access to quality health services. Moreover, co-existing health conditions linked to social disadvantage also magnified the severity of COVID-19 [1, 4].

This was noteworthy in Latin America, where almost 80% of the population lives in cities [6] and deep-rooted inequalities affect cities in this region. Countries as Argentina, that went through a long and strict lockdown, as a mean of avoiding healthcare services collapse [7], showed a greater impact on slums dwellers or other lower income groups [8]. These spatial differences could be explained by contextual characteristics where social distance and isolation were difficult to maintain over time. Aside from material deprivation, other explanations include labour informality, dependency on public transport, and limited possibilities to work from home [9, 10].

Studies assessing the impact of social inequities in COVID-19 incidence, hospitalization, and mortality in small areas within cities have been documented in high-income countries [11–16], but they are not common in middle-income countries, partly due to challenges in obtaining COVID-19 data at small levels of disagregation such as neighborhoods. Nonetheless, these studies have demonstrated a strong association between socioeconomic vulnerability and the degree of COVID-19 severity and death [1, 8, 17–20].

Researchers have widely documented that the risk of developing severe complications and dying from COVID-19 increases sharply with age [21]. However, the magnitude differs across countries: younger people are more likely to die from COVID-19 in middle-income countries compared to high-income countries [21, 22]. Some explanations of these differences may be that the young population in middle-income countries has a higher prevalence of pre-existing diseases, a higher proportion of informal employment, and higher overcrowding, the latter disproportionately affecting the youngest and also related to a more rapid spread of the disease [21].

Research on social inequalities and the differential impact of the pandemic in Latin American cities are more focused in comparing relatively large areas, which may hide meaningful differences across neighborhoods. The development of statistical modeling techniques, such as Bayesian modeling, has facilitated the investigation of the relationships between small areas and helped obtain reliable estimates, particularly when working with small numbers in small areas such as neighborhoods [23–25].

Studies focused on small areas could help policymakers to better understand how these health events are distributed and clustered within cities and target effective interventions at microsocial levels to improve health. Therefore, our aim was to analyze spatial patterning of COVID-19 death rates in neighborhoods in Bariloche city and describe its relationship with socioeconomic characteristics.

## Methods

### Study setting

We conducted this study as part of the SALud URBana en America Latina (SALURBAL) project [26]. The study area was Bariloche, a city of 134,000 residents in 2018, located in the southern region of Argentina, surrounded by a national park with large green areas, part of which is along the southern margins of the Nahuel Huapi Lake. Known worldwide as a tourist attraction, it is home to nearly half of its population lives in neighborhoods with poor and critical living conditions and high income inequalities [27].

### Design and data sources

We performed an ecological study of 159 census tracts from Bariloche, known locally as *radios censales*. Census tracts, henceforth neighborhoods, are the smallest geostatistical census units defined by the national statistics bureau (INDEC) composed by about 300 households. We used confirmed deaths of COVID-19 that were systematically reported to the National Health Surveillance System (SNVS for its acronym in Spanish). These information was complemented by local authorities through the systematic review of other registries, such as civil registration and hospital records [28]. Each record relates to a single death containing information such as age and place of residence. We obtained data from the 2010 census to proxy socioeconomic characteristics of neighborhoods, compiled a by the SALURBAL project.

### Outcome

The outcome was counts of COVID-19 deaths among residents of Bariloche city, spanning the period between June 2020 (first COVID-19 death in Bariloche) and May 2022. Deaths were georeferenced using the street address shapefile from OpenStreetMap in QGIS 3.18® and geocoded to the corresponding census tract using the shapefile of polygons provided by the INDEC [29]. A total of 1.9% of COVID-19 deaths (10 out of 529) could not be georeferenced and were excluded from the analysis. Confidentiality has been protected according to the Protection of Personal Data Law, No. 25,326, and the use of Sensitive Data and the Statistical Confidenciality Law, No. 17.622. Individual information was only used for georeferenciation; all data for analyses was de-identified and aggregated as counts of deaths by age-groups per neighborhood.

We obtained the 2010 census population for each of the neighborhoods by age from REDATAM (acronym of REtrieval of DATa for small Areas by Microcomputer), a software developed by CELADE used in Latin America for processing census data [30]. We also obtained the projected population estimates for 2018 (details in supplementary material, Appendix 1), the latest year for which the data was available, by age at the city level (not by neighborhood) from *Dirección de Estadística y Censos* for the Río Negro province [31]. Applying the age-and neighborhood-specific population proportion from the census 2010 population to the projected 2018 population, we approximated the age-and neighborhood-specific population for 2018, as done in prior studies examining small-area inequalities in mortality in other cities of Argentina [32, 33]. Because our study window spans 2 years, we doubled the estimated 2018 population by age and neighborhood as the population denominator in calculating mortality rates.

Given the role of age in determining mortality, we estimated both crude and age-adjusted death rates due to COVID-19 cumulatively through the end of the study period. We performed direct age-adjustment using the WHO world standard population 2000–2025 [34].

### Exposures

The key exposure variable is the proportion of households in the neighborhood with unmet basic needs, a measure of poverty used in Latin America based on census data [35]. This variable is defined as the percentage of the households with at least one of the following indicators: (a) households with critical overcrowding (3 or more people per room) (b) inconvenient housing units (defined as tenancy piece, precarious households or other type) (c) housing with inadequate services (defined as lack of toilet) (d) households with school-aged (6 to 12 years) children not attending school, and (e) households with high economic dependence (households which have 4 or more individuals per working member and with the household head who has not completed the third grade of primary school) [36].

In addition, we also included the following measures of area-level socioeconomic status (SES): completed high school (% of the population aged 25 years and above with completed high school), piped water inside the dwelling (% of households with piped water inside the dwelling), overcrowding (% of households with more than 3 person per room), school attendance (% of population aged 15 to 17 years attending school), sewage connection (% of households connected to a sewage system of any type), and unemployment rate (% of the population 15 years or older who are unemployed and are actively seeking employment).

These variables were selected since they have been associated with other outcomes in small areas in Latin American cities in previous studies [27, 32, 33, 37, 38], are good measures of area-level socio-economic status (SES), are directly linked to COVID-19 transmission [18], and are commonly available from census data at small area level. They were all retrieved from the 2010 census [30].

### Statistical analysis

We aggregated the deaths in each neighborhood into 4 age groups (0–39 years, 40–59 years, 60–79 years, 80 years and above) and modeled the age-specific mortality rates via a Poisson model with the number of deaths by age group as the outcome and population by age group as the offset. We used a Bayesian approach, accounting for spatial dependency, which enables the borrowing of information from neighboring areas and is particularly useful in obtaining reliable estimates when the number of events (sample sizes) is small [39].

Specifically, we modeled the number of COVID-19 deaths in age group $a$ for small area $i$, denoted ${y}_{ia}$, as being Poisson-distributed with mean ${n}_{ia}{\uplambda }_{ia}$, where ${n}_{ia}$ and ${\uplambda }_{ia}$ denote the population size and COVID-19 mortality rate, respectively. To model ${\uplambda }_{ia}$, we assume:

$$log{\uplambda }_{ia}={ \beta }_{0a} +{z}_{i} +{\phi }_{ia},$$

where ${\beta }_{0a}$ are the age-specific intercepts corresponding to the overall log mortality rate in each of the four age groups, ${z}_{i}$ is spatially structured random effect (clustering) modeled by conditional autoregressive (CAR) distribution and ${\phi }_{ia}$ is an unstructured, zero-mean random effect (heterogeneity). We assigned vague, uninformative priors with large variances for each of the parameters of interest, thereby allowing inference on these parameters to be driven primarily by the data.

The models were run using the R2WinBUGS package in R [40] and 4,000 iterations’ worth of samples were obtained for inference after first discarding pre-convergence samples as *burn-in* and thinning samples to reduce autocorrelation (Software code available in supplementary material, Appendix 2). We then used the posterior samples of age-specific mortality rates derived from the model to calculate crude and age-adjusted rates. To obtain the crude rates for each neighborhood, we aggregated the posterior samples of age-specific mortality rates based on the age distribution within the neighborhood. For age-adjusted rates, we used the population weights from the WHO world 2000–2025 age distribution. As a result, we obtained 4000 estimates of crude- and age-adjusted mortality rates for each neighborhood. We present the median of these estimates in a choropleth map of the crude and age-adjusted mortality rates.

To study the association between age-adjusted mortality rates and socio-economic characteristics of the neighborhoods, we fit two sets of simple linear regression models (a) with the logarithm of the age-adjusted mortality rate as the outcome and (b) mortality rate per 10,000 as the outcome. We used seven predictor variables that proxy the socio-economic context of neighborhoods, one at a time, for easier interpretation. We parameterized explanatory variables by creating the deciles of each of them and re-coding the value of the exposure variable on a 0–1 scale, such that values within the 1^st^ decile are recoded to 0, those in the 2^nd^ decile are recoded to 1/9, and so on. The estimated slope from regression analysis with this parameterization of explanatory variables and mortality rate as the outcome variable is the slope index of inequality (SII), a measure of the absolute inequality in the mortality rate between areas with high values of explanatory variables (9th decile) and areas with low values of explanatory variables (1^st^ decile), while the model with log mortality rate as the outcome variable results in the relative index of inequality (RII), a relative inequality (ratio) measure. We ran the regression analyses for each of the 4000 samples and pooled the regression coefficients and standard errors with Rubin’s formula [41], using mitlml R package.

Bivariate choropleth maps were created to visualize associations between the outcome (age-adjusted COVID-19 mortality rates) and each socio-economic characteristic.

## Results

From June 2020 to May 2022, a total of 529 COVID-19 deaths were registered in Bariloche. The median number of deaths and population per neighborhood were 3 and 789, respectively. A median of 90.0% (10^th^ percentile(p10)- 90^th^ percentile (p90): 78.4–100) of the population aged 15 to 17 years was attending school, but a lower proportion (median 55.9%; p10-p90 25.6–82.9) of individuals over 25 years completed high school. A median of 4.8% (p10-p90: 0.6–22.7) of the population had unmet basic needs and 94.3% (p10-p90: 58.6–100) of the households were connected to a sewage system revealing considerable heterogeneity between neighborhoods (Table 1).

**Table 1:** COVID-19 mortality indicators and socioeconomic characteristics in neighborhoods of Bariloche, Argentina (*n* = 159)

Characteristics	Median	(10^th^, 90^th^ percentile)
Number of deaths^a^	3	(1, 6)
Total population^b^	789	(459, 1335)
Population over 65 years^b^	67	(31, 124)
COVID-19 crude death rate, unmodeled (per 10,000)	17.9	(6.3, 35.2)
COVID-19 crude death rate, modeled (per 10,000)	18.4	(10.5, 30.2)
COVID-19 age-adjusted COVID-19 death rate (per 10,000)	14.8	(11.0, 24.9)
Unemployment rate^c^	6.1	(2.8, 11.3)
% of population over 25 years who completed high school	55.9	(25.6, 82.9)
% of population aged 15–17 attending school	90.0	(78.4, 100)
% of households with overcrowding^d^	1.2	(0, 6.7)
% of households with at least one unmet basic needs^e^	4.8	(0.6, 22.7)
% of households connected to a sewage system of any type	94.3	(58.6, 100)
% of households with piped water access inside the dwelling	98.1	(87.2, 100)

^a^COVID-19 deaths between June 2020 and May 2022

^b^Population for year 2018

^c^Unemployment: % among the population 15 years or above who are not employed and are actively seeking work

^d^Overcrowding: % of households with more than 3 people per room

^e^Unmet basic needs: proportion of households with at least one of the following conditions: critical overcrowding, inconvenient housing units, inadequate services, school-age children not attending school, and high economic dependence (for more details, see *Methods*)

**Fig. 1:**
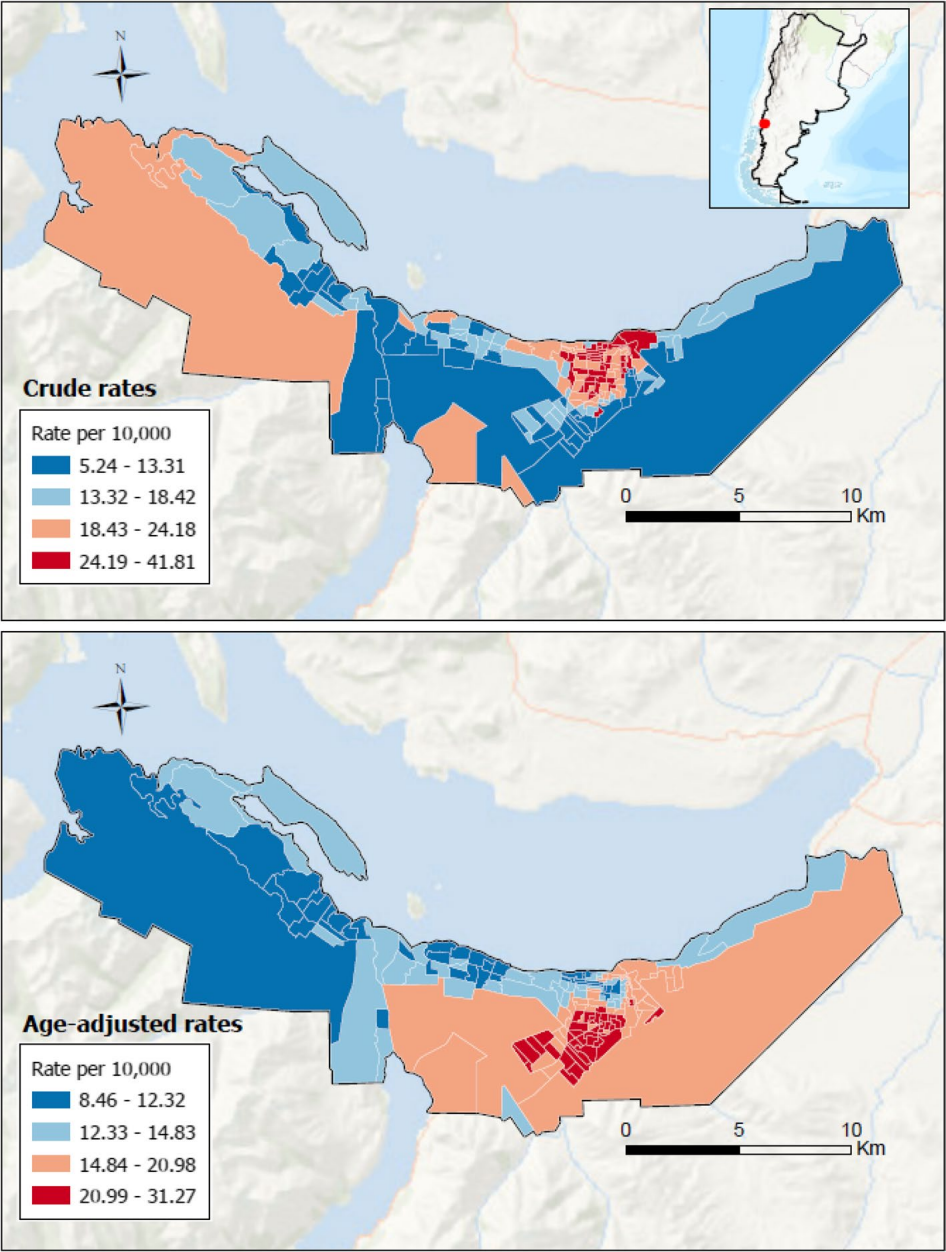
Crude and age-adjusted COVID-19 mortality rate per 10,000. Bariloche, Argentina. June 2020—May 2022. Footnote: legend has different cutpoints since crude and age-adjusted mortality rates are not directly comparable with each other. The box in the upper right margin refers to Argentina, in this map the Argentine Antarctica was omitted for graphic purposes

Figure 1 displays maps of modeled crude (top panel) and age-adjusted (bottom panel) COVID-19 death rates, obtained from Bayesian spatial modeling. Crude death rates across neighborhoods ranged from 5.2 to 41.8 deaths per 10,000 inhabitants, and age-adjusted rates from 8.5 to 31.3 per 10,000 inhabitants (Fig. 1). Death rates were not randomly distributed showing a spatial pattern with clear clustering within the city. The crude death rates were higher in the core area of the city extending to the immediate western neighborhoods. In contrast, the distribution of age-adjusted mortality rates was a mirror image of the crude rates, with higher rates in the southeastern neighborhoods, and lower rates in the core area and westward in the neighborhoods along the shoreline.

**Fig. 2:**
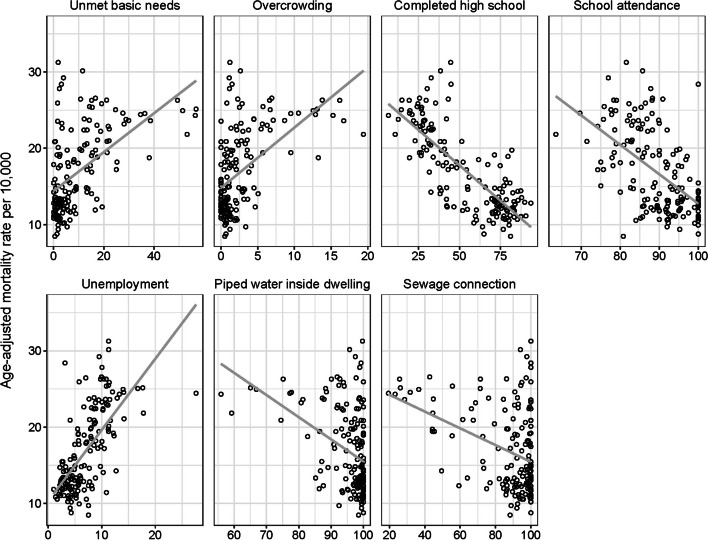
Scatterplot for the association between neighborhood socioeconomic indicators and age-adjusted COVID-19 age-adjusted mortality rate. Bariloche, Argentina. June 2020—May 2022

We found that all SES variables examined were associated with age-adjusted COVID-19 mortality rates: neighborhoods with lower SES had higher COVID-19 death rates than those with higher SES. Figure 2 shows the correlation between each socioeconomic measure and the age-adjusted mortality rate. As expected, education, sewage, and water inside the dwellings were negatively associated with the outcome (higher values in each exposure, which imply higher SES, were associated with lower rates). On the other hand, overcrowding, unemployment, and unmet basic needs were positively associated (higher values in each of these exposures, implying lower SES, were associated with higher rates).

**Fig. 3:**
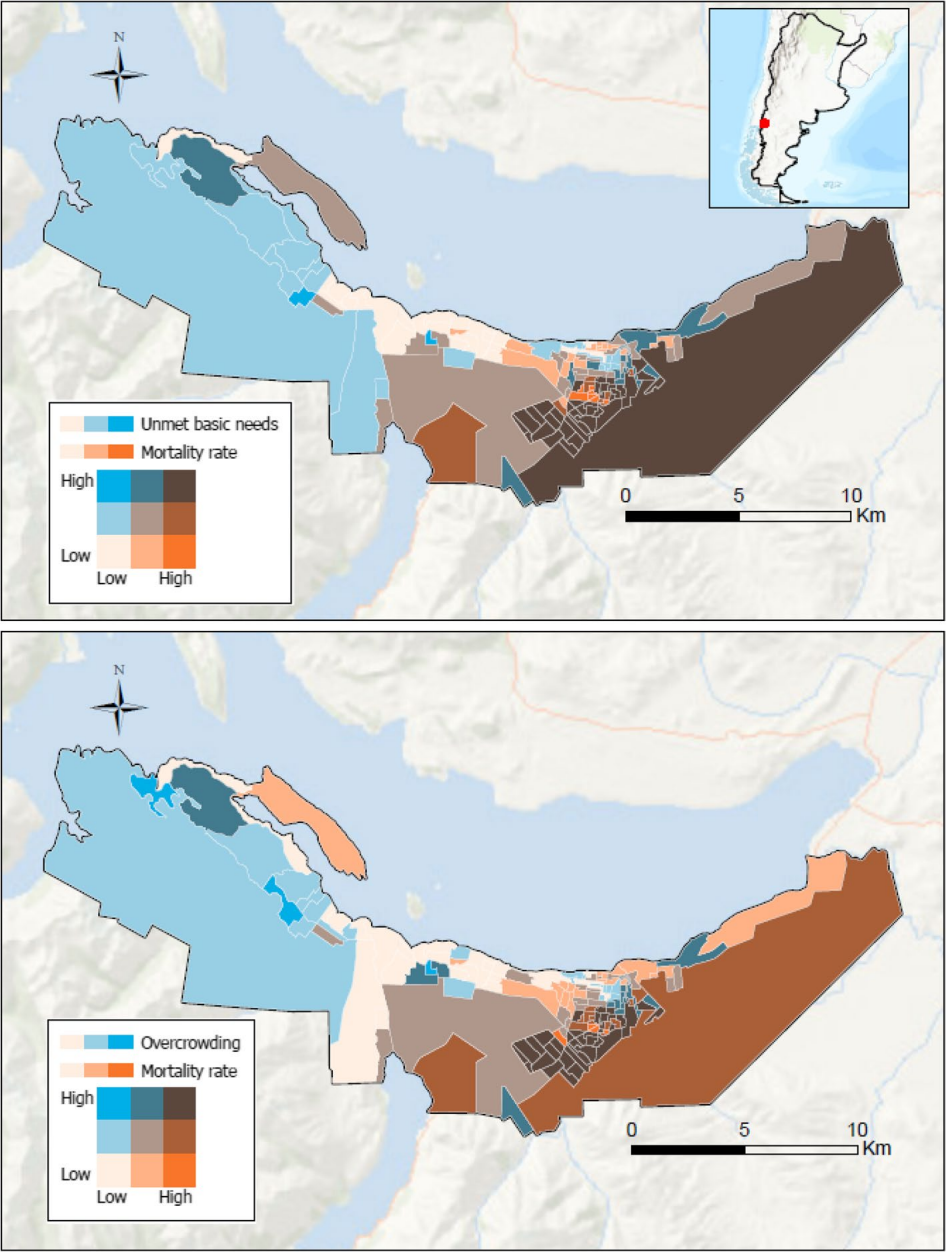
Spatial distribution of COVID-19 age-adjusted mortality rates and selected socioeconomic variables in Bariloche, Argentina. Footnote: In these maps the darker the light blue the higher the percentage of population with unmet basic needs or with overcrowding, and the darker the orange, the higher the age-adjusted COVID-19 mortality rate. The bivariate pallet shows areas of high concordance in brown and in light orange. Brown (high/high in the legend) represents the most unequal areas, *high* COVID-19 age-adjusted mortality rates and more deprived areas (*high* unmet basic needs or overcrowding); light orange in the other extreme (low/low in the legend), *low* COVID-19 age-adjusted mortality rates and less deprived areas (*low* unmet basic needs or overcrowding). The box in the upper right margin refers to Argentina, in this map the Argentine Antarctica was omitted for graphic purposes

The spatial patterning of the different SES measures presented a similar pattern to the spatial distribution of age-adjusted COVID-19 mortality (eFigure 1). The bivariate maps in Fig. 3 simultaneously show the spatial distribution of COVID-19 age-adjusted mortality rates and the two selected SES characteristics (unmet basic needs and overcrowding). The rest of the exposures are available in supplementary material (eFigure 2). We were able to identify two areas of spatial concordance in the city. One area in the southeastern neighborhoods with high mortality and lower SES, and an opposite one, in the core area and western neighborhoods, with lower mortality and higher SES.

Table 2 shows the magnitude of these associations. Education, sewage and water indicators were reverse-coded to facilitate comparison with other indicators. In general, we found that people living in the most deprived areas presented higher mortality rates than those living in the least deprived areas. All variables, except for sewage connection, exhibited a statistically significant relationship with the age-adjusted mortality rate. The SII (difference in mortality rate between the 9^th^ and 1^st^ decile of each variable) ranged from a low of 4.83 (95% CI -0.87 to 10.54) for sewage connection to a high of 13.39 (95% CI 7.17 to 19.60) for completed high school education. The RII (ratio of mortality rates between the 9^th^ and 1^st^ decile of each variable) ranged from a low of 1.31 (95% CI 0.95 to 1.78) for sewage connection to a high of 2.14 (95% CI 1.55 to 2.96) for completed high school education.

**Table 2:** Slope index of inequality and Relative index of inequality in age-adjusted COVID-19 death rate associated with selected socioeconomic indicators

	**SII**^**a**^** in mortality rate per 10,000 (95% CI)**	**RII**^**b**^** in mortality rate (95% CI)**
% of population with at least one unmet basic need	9.77 (4.05,15.49)	1.77 (1.31,2.40)
% of households with overcrowding	9.96 (4.23,15.68)	1.74 (1.29,2.33)
% of population over 25 years who did not complete high school^**c**^	13.39 (7.17,19.60)	2.14 (1.55,2.96)
% of population aged 15–17 years not attending school^**c**^	10.04 (4.65,15.43)	1.77 (1.33,2.36)
Unemployment rate	12.12 (6.26,17.99)	1.98 (1.46,2.69)
% of households not connected to a sewage system of any type^**c**^	4.83 (-0.87,10.54)	1.31 (0.96,1.78)
% of households without piped water access inside the dwelling^**c**^	7.45 (1.96,12.94)	1.53 (1.14,2.05)

Models were run separately, one variable at a time, and were not adjusted for any other variable; CI = Confidence Interval

^a^SII = slope index of inequality, or the difference between the mortality rates of neighborhoods at the 9^th^ vs 1^st^ decile of each socioeconomic variable

^b^RII = Relative index of inequality, or the ratio between the mortality rates of neighborhoods at the 9^th^ vs 1^st^ decile of each socioeconomic variable

^c^These variables are reversed so that the 9^th^ decile represents the most unfavorable socioeconomic conditions

## Discussion

### Summary of the main results

We used georeferenced death records to characterize urban inequalities in COVID-19 mortality in a medium-sized Latin American city. We found important spatial patterning of COVID-19 mortality and also found that this spatial patterning was closely linked to social conditions. People living in the most deprived areas had between 1.3- and 2-times higher COVID-19 death rates than those living in the least deprived areas, depending on the SES characteristic studied. Studies of spatial variation in health outcomes across small areas can shed light on the presence and magnitudes of health inequities [42].

### Spatial distribution

We found that COVID-19 mortality rates were not randomly distributed across the city. Spatial modeling provides us with a means to detect and quantify patterns and investigate the relationship between potential exposures with the studied outcome [43]. In addition to identifying strong spatial patterning, we also documented a strong association of spatial differences with social differences. Hence, space becomes a privileged analytical category due to its potential to mediate social dimensions and health, since it is where social relations produce and reproduce [44].

We were able to both identify the spatial patterning of COVID-19 mortality and quantify its relationship with social factors, identifying a clear social gradient: the more deprived the area of residence, the higher the mortality rates. Different measures of SES were included because each one captures a different dimension and may reflect different pathways linking social conditions to COVID-19 although they are all interrelated. Specifically, educational level and unemployment showed the strongest association with COVID-19 mortality rates. Lower educational level and higher unemployment are related to poorer working conditions which may favor virus transmission by impacting the ability to stay home for fear of being fired, the lack of paid sick leave, work in the informal economy, or work in jobs that do not facilitate working from home or social distancing [13, 45]. Unmet basic needs and overcrowding showed a slightly weaker but still substantial association with COVID-19 deaths. These variables proxy precarious living conditions, which make social distancing physically impossible since multiple persons must share the same room. Poor quality housing is associated with higher risks of mortality from COVID-19 and these are more likely to be located in deprived areas and inhabited by people with lower incomes [45].

The consistency with different SES measures linked to COVID-19 deaths lends strength to the idea that social inequality is a key driver of COVID-19 mortality. Each indicator is correlated with many other population attributes and may act through many interrelated mechanisms [46]. Pre-existing social and health conditions have a substantial impact on mortality rates from COVID-19 [4, 45]. People from more disadvantaged social classes have a higher prevalence of chronic conditions (hypertension, cardiovascular diseases, diabetes, etc.) [37, 47] and are therefore more vulnerable to severe COVID-19. Deprivation-related inequalities in the mortality rate from COVID-19 showed a pattern that was similar to that observed for mortality from other causes across Latin American cities and across larger areas within cities [38, 48]. Moreover, as in this study, area-level education for small areas was also the indicator with the strongest association with life expectancy across neighborhoods in CABA and Córdoba, two other Argentinian cities [32, 33].

An interesting finding of our study is the impact that age-adjustment had on the spatial patterning of risks of death. Before age-adjustment, death rates were higher in the least deprived areas of the city, but this changed dramatically after age adjustment. The risk of dying from COVID-19 is strongly related to age, with higher mortality rates at older ages. Consequently, the rates in the two areas may appear different, but this may be due to different age distributions rather than to a difference in the underlying risk of COVID-19. Age-adjustment is necessary to accurately describe differences in risks that are unrelated to the age distribution such as those conferred by social conditions. Social conditions may also interact with age. For example, younger individuals in poorer neighborhoods may be at a substantially higher risk of being exposed to COVID-19 infection than younger individuals in less deprived neighborhoods because of the type of jobs they have access to (informal economy, inability to isolate). In addition, because they may be more likely to reside in multigenerational and overcrowded households, they may be more likely to transmit infection to older relatives thus magnifying transmission and severity with implications for mortality rates [21].

### Strengths and limitations

This study has several strengths. First, the availability of geo-referenceable individual-level data on COVID-19 deaths over two years, with less than 2% missing georeferencing. To our knowledge, this is the first study examining COVID-19 data at the neighborhood level in a mid-sized Argentinian city. Second, we were able to conduct direct age-standardization of the outcome, an analysis that is often lacking in COVID-19 research due to the lack of age-specific mortality data. Age-standardization allowed us to uncover a substantially different spatial pattern. The COVID-19 pandemic has highlighted the importance of descriptive epidemiology in responding to serious public health crises, and the challenges in characterizing basic epidemiological metrics [49, 50]. Rigorous descriptive analysis may provide valuable insights into understanding the social patterning of health fundamental to discuss how social organization shapes the distribution of health and disease [46]. Moreover, descriptive studies at small areas reveal disparities in indicators that are obscured when using larger units. This allows for a more precise description of the social reality of an area [44], and the potential identification of vulnerable groups for targeted interventions.

Our study has several limitations. First, small numbers can result in imprecise (statistically unstable) estimates for accurate comparisons [43]. We addressed this limitation by employing a spatial Bayesian framework that smooths mortality rates by borrowing information from age groups and neighboring areas to improve the precision of local estimates, resulting in the smoothing of extreme values [23, 39]. Another limitation was the impossibility of stratifying the analysis by gender due to the low number of deaths, even though gender is an important dimension that would have presented differential risks [51, 52]. Finally, the social characteristics of neighborhoods rely on latest available census (year 2010) and therefore did not align with the period of the pandemic. Unfortunately, the closest census in time (2022) does not yet have available data at the neighborhood level. Oiur analysis therefore relies on the assumption that the relative ranking of social characteristics by neighborhood has remained stable over time. This assumption was studied in previous work for other countries from SALURBAL project, and found that socioeconomic rankings did not change much across time [48].

### Policy recommendations

Studies comparing small areas such as neighborhoods can inform placed-based interventions as well as broader policies aimed at addressing structural drivers of health inequities. Identifying areas where inequalities are combined can target resourses to improve the situation of those at higher risk. Systematic geocoding of all deaths would allow cities to describe and track inequities, and to identify possible pathways for developing effective policy actions within and outside the healthcare system, allowing contextualized problems to be identified and quantified. We should use the accumulated knowledge gained throughout the pandemic to improve the health of populations, particularly those living in the most disadvantaged areas.

### Conclusions

The COVID-19 pandemic has not only exposed the underlying inequalities in society but has magnified them [45]. The most vulnerable populations, such as those living in urban slums and informal settlements, were the worst affected by the immediate and long-term effects of the pandemic [4]. Deep-rooted inequalities in cities, manifested in overcrowded households and unsafe working conditions, made it almost impossible to comply with the lockdowns and stay-at-home measures.

The spatial differences that we observed are driven by the embodiment of historical and social context [34]. Spatial emplacement and segregation are linked to social status, ethnocultural background, and the interrelated effects of inequality and racism, as it happens in Bariloche and other cities. The associations found in the spatial distribution of mortality from an acute illness, such as COVID-19, are possibly related to a long-term historical process, given that the social processes leading to health differences based on social position, race or inmigration involve multiple pathways and long casual chains [46].

### Supplementary Information

**Additional file 1. **

**Additional file 2: eFigure 1.** Spatial distribution of socio-economic variables in Bariloche, Argentina. Note: Data from 2010 census. Legends show the quartiles of the variables (in percentages). Definition of each of these variables are provided in the exposure subsection of the methods section in the main manuscript. In purple scale maps, higher percentages indicates a better socioeconomic characteristics. In orange scale maps, higher percentages indicates a worse socioeconomic characteristics.

**Additional file 3: eFigure 2.** Spatial distribution of COVID-19 age-adjusted mortality rates and socioeconomic variables in Bariloche, Argentina. A first set of bivariate maps with the spatial distribution of COVID-19 age-adjusted mortality rates with the two measures of education (school attendance and completed high school), piped water, or sewage. These maps are in a purple/light blue/green range, where darker the light blue, better the SES (higher education, piped water, or sewage), and darker the green, higher the mortality rates. In the bivariate map, the extremes of high concordance areas are dark-green and light blue. Dark green represents the most unequal areas (high/low in the legend), with *high* COVID-19 age-adjusted mortality rates and more deprived areas: *low* percentage of education, piped water, and sewage. Light-blue areas (low/high in the legend), the other extreme, *low* COVID-19 age-adjusted mortality rates and less deprived areas: *high* percentage of education, piped water, and sewage. In the botton, a map, with the spatial distribution of COVID-19 age-adjusted mortality rates with unemployment, same colours and interpretation as Figure 3. These maps is in a range of brown/light blue/orange colors, where the darker the light blue the worse the SES (higher the unemployment), and the darker the orange, the higher the mortality rate. The bivariate pallet shows areas of high concordance in brown and in light orange. Brown (high/high in the legend) represent the more unequal areas, *high* COVID-19 age-adjusted mortality rates and more deprived areas (*high* unemployment); light orange in the other extreme (low/low in the legend), *low* COVID-19 age-adjusted mortality rates and less deprived areas (*low* unemployment).

## Data Availability

This data cannot be shared openly. The data use agreement does not allow us to share the vital registration data openly. For the rest of the data, we are preparing an open data platform that is planned to be launched in late 2023. The SALURBAL project welcomes queries from anyone interested in learning more about its dataset and potential access to data. To learn more about SALURBAL’s dataset, visit https://drexel.edu/lac/ or contact the project at salurbal@drexel.edu.
