# Supplementary material for: Social inequalities and COVID-19 mortality between neighborhoods of Bariloche city, Argentina
Source: Int J Equity Health. 2023 Sep 28;22:198. doi: 10.1186/s12939-023-02019-w (PMC10537962; doi:10.1186/s12939-023-02019-w)
Supplement: Supplementary file 1 — Additional file 1. [file 12939_2023_2019_MOESM1_ESM.docx]

**Supplementary materials**

**Appendix 1**

Specifically, if *P_2018; as_* represents the projected 2018 age- and sex specific population for the municipality and if *C_2010; asi_* represents the census 2010 age-, sex-, and neighborhood specific population for age *^a={0,1,..,85+}^*, sex *^s={1,2}^* and neighbohood *^i={1,2,..,Ni}^*, then  we estimate the age- sex-, and neighborhood specific population for 2018 as *N*_2018;_*_asi_* =  *P*_2018;_ *_as_* $\boldsymbol{x}\frac{\boldsymbol{c}_{\boldsymbol{2010;asi}}}{\sum_{\boldsymbol{i=1}}^{\boldsymbol{i=}\boldsymbol{N}_{\boldsymbol{i}}} \boldsymbol{c}_{\boldsymbol{2010;asi}}}$

**Appendix 2**

<https://github.com/Binod-Acharya/Bariloche-Covid19-Inequality/blob/main/bariloche_covid_analysis_for_github.R>
