# Supplementary figures and images for: Social inequalities and COVID-19 mortality between neighborhoods of Bariloche city, Argentina
Source: Int J Equity Health. 2023 Sep 28;22:198. doi: 10.1186/s12939-023-02019-w (PMC10537962; doi:10.1186/s12939-023-02019-w)

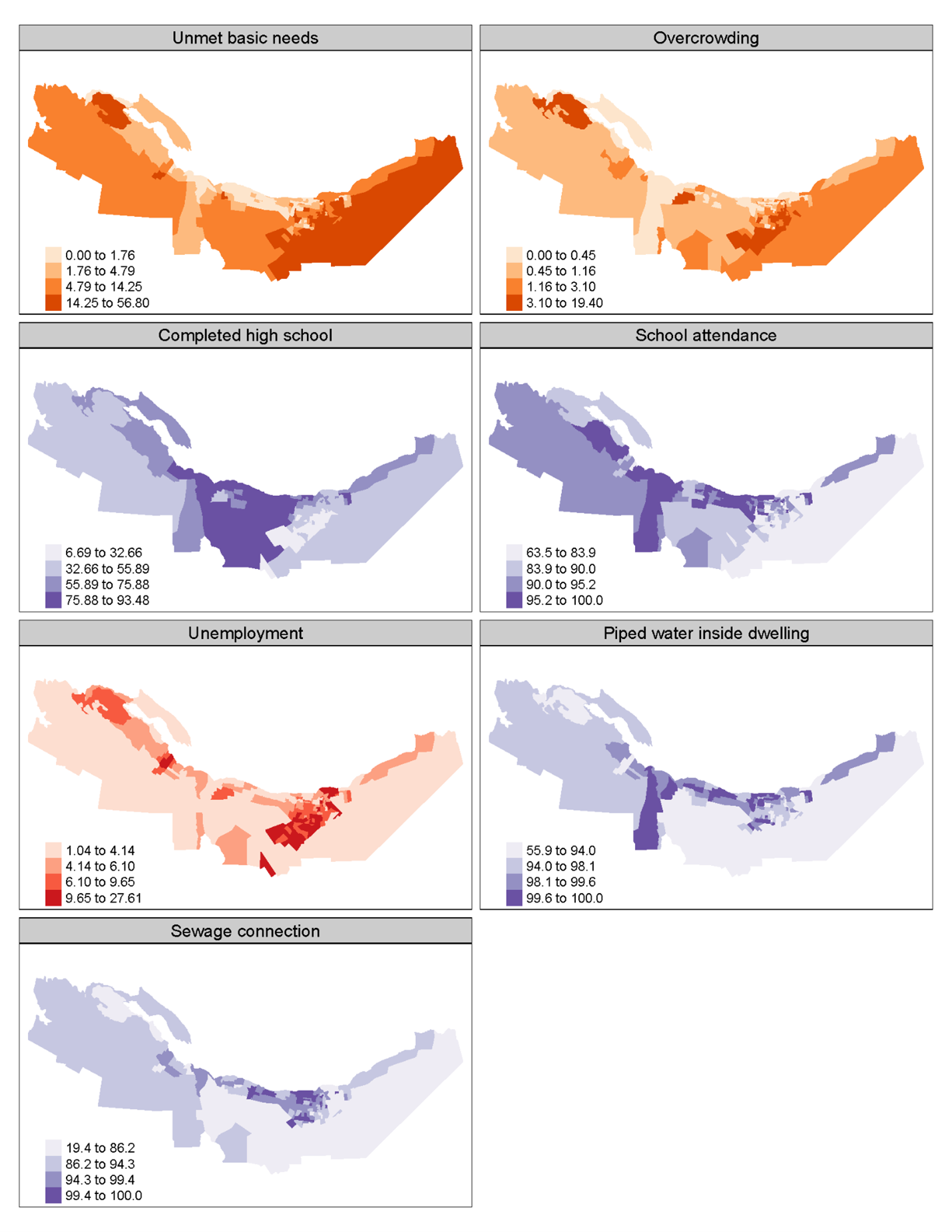

Supplement: Supplementary file 2 — Additional file 2: eFigure 1. Spatial distribution of socio-economic variables in Bariloche, Argentina. Note: Data from 2010 census. Legends show the quartiles of the variables (in percentages). Definition of each of these variables are provided in the exposure subsection of the methods section in the main manuscript. In purple scale maps, higher percentages indicates a better socioeconomic characteristics. In orange scale maps, higher percentages indicates a worse socioeconomic characteristics. [file 12939_2023_2019_MOESM2_ESM.tif]
